# Non-Invasive Respiratory Support: How to Get It Right in Clinical Medicine

**DOI:** 10.3390/jcm12165243

**Published:** 2023-08-11

**Authors:** Stefano Nava

**Affiliations:** 1 Respiratory and Critical Care Unit, Policlinico S. Orsola-Malpighi di Bologna, IRCCS Azienda Ospedaliero Universitaria di Bologna, Via Albertoni 15, 40138 Bologna, Italy; stefanava@gmail.com; 2 Department of Medical and Surgical Science (DIMEC), Alma Mater Studiorum University of Bologna, Via Massarenti 9, 40138 Bologna, Italy

It is with great pleasure and enthusiasm that I introduce this Special Issue of the *Journal of Clinical Medicine*, entitled “Non-invasive Respiratory Support: How to Get It Right in Clinical Medicine”. As the editor, I have had the privilege of collaborating closely with esteemed researchers and clinicians in the field to bring forth a collection of articles that encapsulate the latest advancements, insights, and challenges in the realm of non-invasive respiratory support.

The management of respiratory failure has long been a complex challenge in critical care medicine. Historically, invasive mechanical ventilation was the primary modality employed to support patients with acute or chronic respiratory insufficiency. However, non-invasive respiratory support (NRS) has emerged as a viable alternative in select patient populations, and this held particularly true during the last SARS-COVID-19 pandemic, when the use of NRS even outside a “protected” environment was dramatically increased [1]. This Special Issue aims to explore the various aspects of non-invasive interventions, from their physiological basis to their clinical applications and outcomes.

The articles presented in this collection cover a wide range of topics, encompassing both the fundamental principles and the practical implications of NRS. We begin by delving into the physiological mechanisms underlying the effectiveness of non-invasive ventilation (NIV) and high-flow nasal cannula (HFNC) therapy. These mechanistic insights provide a solid foundation for understanding the rationale behind these interventions and their potential benefits in different patient populations.

In particular, **Vega Pittao et al.** [2] described the mechanisms of HFNC in fields that have still not been fully investigated like chronic hypercapnic respiratory failure, while in a sophisticated ex vivo modelling, **Truschel and Polkey** [3] demonstrated that HFNC is as effective for promoting oxygen diffusion as CPAP when oxygen is entrained at the mask and is superior to CPAP where oxygen is entrained at the back of the device. Specifically, the benefit from alveolar expansion from CPAP pressure or increased end expiratory lung volume (EELV) provides no significant benefit to oxygenation in non-atelectatic lungs.

Subsequently, we explore the clinical aspects of non-invasive respiratory support. Our esteemed contributors share their expertise on patient selection criteria, optimal ventilator settings, and the management of complications associated with NIV and HFNC. Moreover, they discuss the integration of non-invasive interventions into the broader context of patient care pathways, emphasizing the need for interdisciplinary collaboration and tailored treatment strategies.

One of the key challenges in the field is the identification of patients who are most likely to benefit from non-invasive respiratory support. In this Special Issue, we are fortunate to have articles dedicated to the prediction of NIV success, and the development of predictive models for outcomes in different clinical scenarios. These contributions open new avenues for personalized medicine and support clinical decision making in an evidence-based manner.

Additionally, this collection highlights the impact of non-invasive respiratory support across various patient populations, including those with chronic respiratory failure, like chronic obstructive pulmonary disease (COPD), and restrictive thoracic disorders. **Carlucci and Fusar Poli,** in their two papers [4,5] dealing with these two pathologies, present compelling data on the efficacy, safety, and long-term outcomes of non-invasive interventions in these settings, underscoring the need for their integration into routine clinical practice. Interestingly, they also provide useful clinical suggestions on how to optimize the ventilation in these two populations.

In keeping with the two previous papers, **Kampelmacher** [6], in a narrative review, discusses the benefits and challenges of both outpatient and home initiation of NIV. He concluded that outpatient or home initiation of NIV is safe, feasible, and not inferior regarding both acceptance and adherence. Also considered are the potential challenges and risks associated with these initiation strategies, including logistical issues, technical problems, complications, and patient selection.

When choosing a ventilator for a home program, there are differences in the details within each model about triggering, pressurization, or autotitration algorithms that are ignored, but these differences are important and may contribute to drawbacks that may occur in individual patients. In their review, **Luján and Lalmolda** [7] describe the systems that popular models of ventilators use for triggering EPAP titration and the response to unintentional leaks.

The selection of circuits, masks, filters, and additional consumables are also critical and insufficiently studied items in the practice of NRS. Another review by **Luján et al.** [8] demonstrates with a suite of certain bench experiments that it is possible to determine the implications each of these selections have in a clinical practice.

**Arnal et al.** [9] tackle the important issue of monitoring NIV. This is required to optimize the ventilator settings when the lung condition changes over time and to detect common problems such as unintentional leaks, upper airway obstructions, and patient–ventilator asynchronies. The review describes the indication, validity criteria, and interpretation of nocturnal oximetry and transcutaneous capnography, the role of polygraphy and polysomnography and recommends the telemonitoring of the ventilator as a useful tool that should be integrated in the monitoring strategy.

Furthermore, we recognize the growing importance of Mechanical Insufflation-Exsufflation (MIE) as a valuable adjunctive therapy in non-invasive respiratory support. **Chatwin and Wakeman** [10] highlighted how MIE provides a unique mechanism of assisting cough and airway clearance, particularly in patients with neuromuscular disorders or compromised airway clearance due to respiratory conditions. Several articles in this Special Issue also explore the applications, techniques, and outcomes of MIE, shedding light on its role in optimizing non-invasive respiratory support.

The landscape of non-invasive respiratory support continues to evolve rapidly, with emerging technologies and novel interventions on the horizon. In this Special Issue, **Prediletto et al.** [11] also explore these exciting developments, such as extracorporeal carbon dioxide removal (ECCO2R), and the role of acute NIV in dramatically reducing the need for ICU admission of COPD patients. These articles not only provide a glimpse into the future but also provoke critical thinking and inspire further research in this vibrant field.

As we navigate the complexities of non-invasive respiratory support, it is crucial to reflect on the challenges and limitations that lie ahead. While these interventions hold tremendous promise, their successful implementation requires a comprehensive understanding of patient phenotypes, careful patient selection, and ongoing monitoring to ensure optimal outcomes. This Special Issue aims to foster discussions and collaborations that push the boundaries of knowledge, paving the way for a future where non-invasive respiratory support becomes the cornerstone of respiratory care.

I would like to express my deepest gratitude to all the authors who have contributed their invaluable research and insights to this Special Issue. Their dedication, expertise, and commitment to advancing the field of non-invasive respiratory support have made this collection possible. Their tireless efforts to unravel the complexities of respiratory failure and improve patient outcomes are commendable.

I would also like to extend my appreciation to the reviewers who have dedicated their time and expertise to ensure the scientific rigor and quality of the articles in this Special Issue. Their thoughtful evaluations and constructive feedback have been instrumental in shaping the final manuscripts and enhancing the overall scientific merit of this collection.

Furthermore, I would like to thank the *Journal of Clinical Medicine* editorial team for their unwavering support and dedication. Their tireless efforts in managing the peer-review process, coordinating with authors and reviewers, and ensuring the smooth publication of this Special Issue have been instrumental.

Finally, I would like to express my gratitude to all the organizations, institutions, and companies that have contributed to the advancements in non-invasive respiratory support. Their ongoing support, research funding, and commitment to innovation play a vital role in shaping the future of respiratory care. While the contributions of all these entities are essential, I would like to extend a special thanks to Breas Medical for their support and collaboration. Their dedication to advancing non-invasive respiratory support aligns perfectly with the objectives of this Special Issue.

In conclusion, I am delighted to present this Special Issue on “Non-invasive Respiratory Support: How to Get It Right in Clinical Medicine”. The articles contained within this collection provide a comprehensive and up-to-date overview of the field, offering insights into the physiological principles, clinical applications, and future directions of non-invasive respiratory support. It is my hope that this compilation will serve as a valuable resource for clinicians, researchers, and healthcare professionals, facilitating the adoption of evidence-based practices and improving the care and outcomes of patients with respiratory failure.
